# Use of serum KL-6 and chest radiographic severity grade to predict 28-day mortality in COVID-19 patients with pneumonia: a retrospective cohort study

**DOI:** 10.1186/s12890-024-02992-0

**Published:** 2024-04-18

**Authors:** Jing Zou, Yiping Shi, Shan Xue, Handong Jiang

**Affiliations:** 1 Department of Respirology, Ren Ji Hospital, Shanghai Jiao Tong University School of Medicine, No.160 Pujian Rd, 200127 Shanghai, China; 2 Department of Nuclear Medicine, Ren Ji Hospital, Shanghai Jiao Tong University School of Medicine, Shanghai, China

**Keywords:** COVID-19, KL-6, HRCT, Mortality

## Abstract

**Background:**

Coronavirus disease 2019 (COVID-19) has had a global social and economic impact. An easy assessment procedure to handily identify the mortality risk of inpatients is urgently needed in clinical practice. Therefore, the aim of this study was to develop a simple nomogram model to categorize patients who might have a poor short-term outcome.

**Methods:**

A retrospective cohort study of 189 COVID-19 patients was performed at Shanghai Ren Ji Hospital from December 12, 2022 to February 28, 2023. Chest radiography and biomarkers, including KL-6 were assessed. Risk factors of 28-day mortality were selected by a Cox regression model. A nomogram was developed based on selected variables by SMOTE strategy. The predictive performance of the derived nomogram was evaluated by calibration curve.

**Results:**

In total, 173 patients were enrolled in this study. The 28-day mortality event occurred in 41 inpatients (23.7%). Serum KL-6 and radiological severity grade (RSG) were selected as the final risk factors. A nomogram model was developed based on KL-6 and RSG. The calibration curve suggested that the nomogram model might have potential clinical value. The AUCs for serum KL-6, RSG, and the combined score in the development group and validation group were 0.885 (95% CI: 0.804–0.952), 0.818 (95% CI: 0.711–0.899), 0.868 (95% CI: 0.776–0.942) and 0.932 (95% CI: 0.862–0.997), respectively.

**Conclusions:**

Our results suggested that the nomogram based on KL-6 and RSG might be a potential method for evaluating 28-day mortality in COVID-19 patients. A high combined score might indicate a poor outcome in COVID-19 patients with pneumonia.

## Background

Coronavirus disease 2019 (COVID-19) is a pandemic disease, and its clinical features are diverse. It induces lung damage by triggering a hyperinflammatory response [1]. COVID-19 patients present mild to severe symptoms and even rapidly develop life threatening respiratory failure. Severe/critical COVID-19 is marked by acute respiratory distress syndrome (ARDS), sepsis, multisystem organ failure, hyperinflammation and other extrapulmonary manifestations [2–5]. Many risk factors have been identified to be putatively relevant to the mortality of COVID-19, including older age, preexisting comorbidities [6, 7], lymphocytopenia, C-creative protein (CRP), lactate dehydrogenase (LDH) and interleukin-6 (IL-6) [8, 9].

Although a few studies were designed to analyse risk factors affecting mortality in COVID-19 patients, clinicians still lack effective tools to predict their short term outcomes, currently. No nomogram model that combined molecular biomarkers representing lung damage and chest radiological features has been developed to predict the 28-day mortality in COVID-19 patients. In this article, we presented a retrospective analysis to establish an easy nomogram model with Krebs von den Lungen-6 (KL-6) and radiographic features to predict 28-day mortality in COVID-19 patients with pneumonia.

## Methods

### Patients

From December 12, 2022 to February 28, 2023, a total of 173 patients met the inclusion criteria: (1) age 18 years and older; (2) diagnosis of COVID-19 was confirmed when RT-PCR of SARS-CoV-2 result was positive; (3) chest HRCT scan confirmed with pneumonia, which was performed less than 24 h before admission or within 24 h after admission in hospital; 3) patients did not present preexisting of interstitial lung disease; and (4) serum KL-6 was tested within 24 h of admission.

The Ethics Committee of Ren Ji Hospital approved the study (LY2023-064-B). The Ethics Committee of Ren Ji Hospital waived the requirement for informed consent of patients due to the retrospective nature of the study and all data were analysed anonymously.

Disease severity for COVID-19 in this study was evaluated by WHO criteria at the time of admission. Severe COVID-19 is defined by any of the following: (1) oxygen saturation < 90% on room air; (2) signs of pneumonia; and (3) signs of severe respiratory distress (in adults, accessory muscle use, inability to complete full sentences, respiratory rate > 30 breaths per minute). Critical COVID-19 is defined by the criteria for ARDS, sepsis shock or other conditions that would normally require the provision of life-sustaining therapies such as mechanical ventilation (invasive or noninvasive) or vasopressor therapy. Nonsevere COVID-19 is defined as the absence of any criteria for severe or critical COVID-19 [10].

### Chest HRCT protocols

All images were obtained on one of the two CT systems (uCT 760, United Imaging, China; Optima 660, GE, America) with patients in a supine position. The main scanning parameters followed the manufacturers’ standard recommended presetting for a thorax routine. Images were reconstructed with a 0.625–1.250 mm slice thickness in all cases.

### Image analysis

Two radiologists (H.W and X.G with 24 and 15 years of experience in interpreting chest HRCT imaging, respectively), who were blinded to the severity and outcome of the disease, reviewed all chest HRCT images and agreed upon by consensus. The radiologists described the main CT features (GGO, consolidation, CPV). GGO, CPV pattern and consolidation were based on the standard glossary for thoracic imaging reported by the Fleischner Society [11]. The radiological severity grade (RSG) was determined based on the percentage of lung involvement in grades 1, 2, 3 and 4, which was 0–25%, 26–50%, 51–75% and 76–100%, respectively.

### Statistical analysis

Continuous variables are expressed as medians and the 25th -75th percentiles of the interquartile range (IQR). The data did not show a normal distribution. The Mann - Whitney *U* test was used to compare pairs of variables. The chi-squared test was used for categorical variables as appropriate. We applied Spearman’s rank correlation to nonparametric data. The Cox proportional hazard model was used to identify the most important factors of 28-day mortality in COVID-19 patients. The synthetic minority oversampling technique (SMOTE) strategy was used to produce synthetic examples to overcome the problem of class imbalance for development group [12].And a nomogram was developed based on the SMOTE strategy (smote group). The concordance index (C-index) was calculated to evaluate the predictive accuracy in both the smote group and validation group. Time-dependent receiver operating characteristic (ROC) curves, including the area under the curve (AUC) and its 95% confidence interval (95% CI), were analysed by the time ROC package to evaluate the performance of prognostic prediction. The optimal threshold for each selected factor and combined score was determined when the Youden index achieved the highest value. DeLong test was performed to compare the difference between two AUCs. On the basis of the optimal cut-off given by the ROC curve analysis, all of the factors under study were transformed to binary variables, and their corresponding cumulative 28-day survival rates were calculated by the Kaplan - Meier method. All statistical analyses were conducted using SPSS version 16.0 and R software (version 4.3.3 (http://www.Rproject.org)). Statistical significance was decided by a criterion of two-sided *p* < 0.05.

## Results

### Patient characteristics

**Fig. 1:**
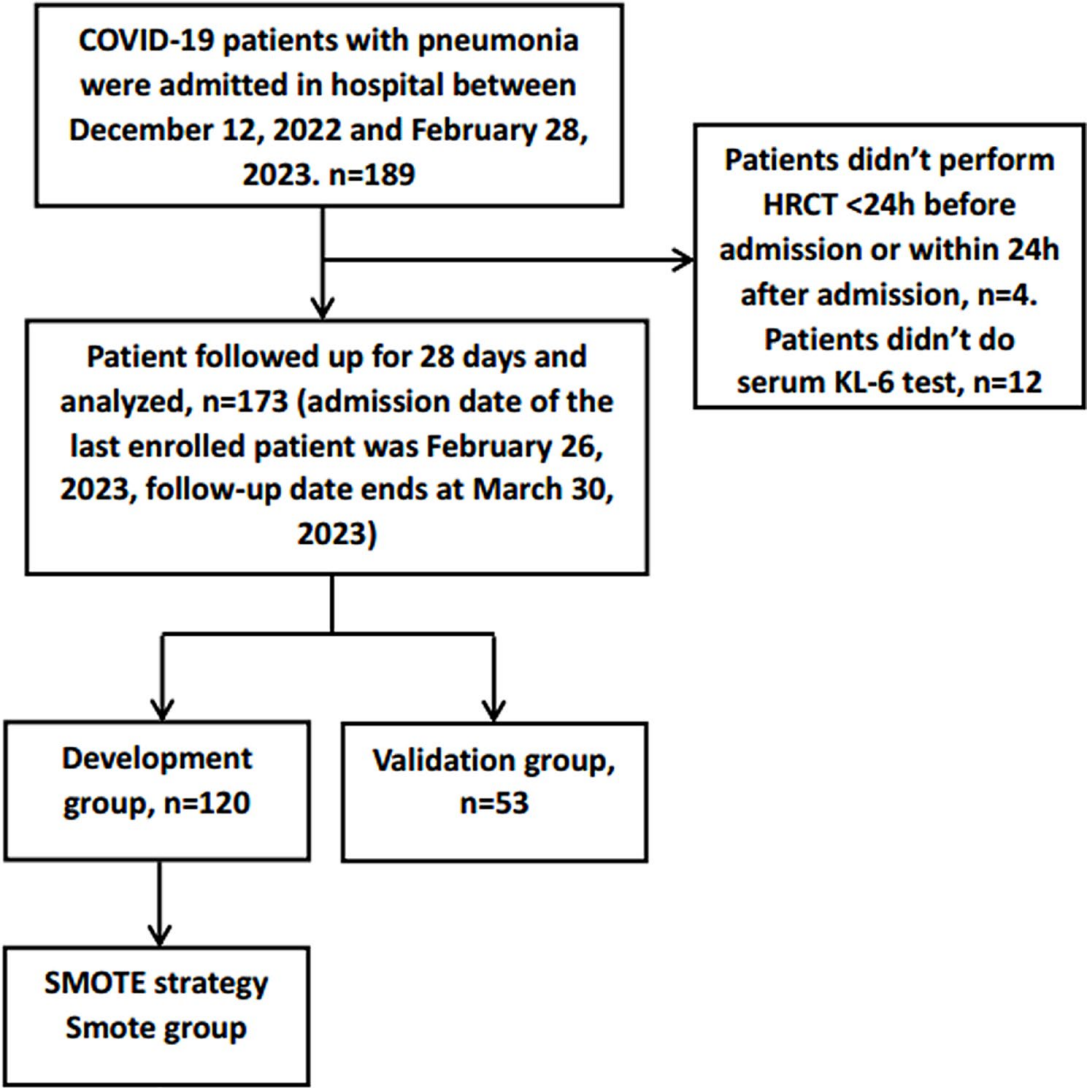
Flowchart of patients

A total of 189 patients with COVID-19 with pneumonia were hospitalized, and 173 patients met the inclusion criteria (Fig. 1). The clinical characteristics and laboratory findings are shown in Table 1 (development group = 120, validation group = 53). All patients were ethnically Asian. The 28-day mortality was 23.7% (41 of 173). The median age of these patients was 74 (range, 23–95 years). A total of 4.6% (8 of 173) of the total patients were younger than 40 years, while 87.9% (152 of 173) were above the age of 60 years. A total of 70.5% were males. The proportion of severe/critical patients was 57.8% (100/173) on admission. The 1st and 2nd most common underlying diseases in our study were hypertension (58.4%, 101/173) and diabetes mellitus (31.8%, 55/173), respectively.

**Table 1:** Main characteristics of COVID-19 patients

Parameters	Development group (*n* = 120)			Validation group (*n* = 53)			Development group vs. Validation group	Total (*n* = 173)
	Survivors (*n* = 93)	Non-survivors (*n* = 27)	*p* value	Survivors (*n* = 39)	Non-survivors (*n* = 14)	*p* value	*p* value	
Age	74(65–81)	76(70–84)	0.150	72(64–78)	77(71–83)	0.075	0.271	74(67–80)
Gender, Male/Female	66/27	20/7	0.813	23/16	13/1	0.022	0.718	122/51
BMI	23.7(21.5–25.9)	23.8(22.5–25.7)	0.811	23.8(20.6–25.8)	24.7(21.5–25.4)	0.535	0.619	23.8(21.6–25.7)
Hypertension, n(%)	49(52.7)	16(59.2)	0.662	25(64.1)	11(78.5)	0.506	0.097	101(58.4)
Diabetes mellitus, n(%)	29(31.1)	9(33.3)	0.819	13(33.3)	4(28.5)	1.000	1.000	55(31.8)
Coronary heart disease	7(7.5)	5(21.7)	0.139	2(5.1)	1(7.1)	1.000	0.558	15(8.7)
Chronic renal disease, n(%)	7(7.5)	6(28.7)	0.071	7(17.9)	2(14.3)	1.000	0.322	22(12.7)
Cerebrovascular disease	6(6.5)	2(7.4)	1.000	2(5.1)	1(7.1)	1.000	1.000	11(6.4)
Alzheimer’s disease, n(%)	1(0.1)	3(11.1)	0.035	2(5.1)	0(0.0)	1.000	1.000	6(3.5)
COPD, n(%)	5(5.3)	2(7.4)	0.654	5(12.8)	2(14.8)	1.000	0.130	14(8.1)
Cancer*	3(3.2)	3(11.1)	0.127	0(0.0)	2(14.8)	0.066	1.000	8(4.6)
Disease Severity (severe/critical), n(%)	40(43.0%)	25(92.6%)	< 0.001	21(53.8)	14(100.0)	0.002	0.182	100(57.8)
Serum KL-6 concentration, U/ml	348(221–535)	969(678–1231)	< 0.001	369(220–500)	1007(784–2095)	< 0.001	0.515	436(252–728)
P/F ratio	321(260–388)	148(105–238)	< 0.001	293(245–347)	163(106–188)	< 0.001	0.068	281(218–357)
NLR	5.6(3.4–11.1)	14.6(8.8–25.5)	< 0.001	7.9(4.1–14.1)	12.4(7.9–55.9)	0.016	0.145	8.1(4.1–13.5)
CRP, mg/dl	37.7(11.4–81.8)	101.9(54.8-193.9)	< 0.001	36.8(20.5–86.5)	71.7(47.3–94.5)	0.060	0.717	50.5(15.7–93.2)
IL-6,pg/ml	12.9(6.6–37.9)	100.2(30.4-283.9)	< 0.001	26.0(10.5–63.8)	125.2(58.9–620.0)	< 0.001	0.030	26.3(8,1-68.9)
ALT, U/L	28(18–40)	28(19–47)	0.410	24(17–44)	24(21–47)	0.627	0.678	27(19–42)
ALB, g/L	33.2(30.4–36.1)	30.3(26.8–33.0)	0.004	32.5(27.9–35.8)	31.2(28.1–33.1)	0.336	0.383	32.5(29.1–35.2)
LDH, U/L	287(224–355)	511(356–590)	< 0.001	318(249–420)	336(202–507)	0.725	0.545	312(238–414)
BUN, mmol/L	7.6(4.7–9.5)	9.9(6.3–21.6)	< 0.001	7.7(5.6–11.0)	8.5(5.2–13.0)	0.679	0.515	8.0(5.3–10.9)
Scr, µmol/L	74(56–91)	80(67–156)	0.052	79(63–106)	64(55–133)	0.679	0.729	75(60–104)
Radiological features								
RSG			< 0.001			< 0.001	0.723	
Grade 1, n(%)	31(33.3)	1(3.7)		14(35.9)	0(0.0)			46(26.6)
Grade 2, n(%)	39(41.9)	5(18.5)		16(41.0)	2(14.3)			62(35.8)
Grade 3, n(%)	20(21.5)	9(33.3)		6(15.4)	7(50.0)			42(24.3)
Grade 4, n(%)	3(3.2)	12(44.4)		3(7.7)	5(35.7)			23(13.3)
Pure GGO/ GGO + Consolidation	43/50	9/18	0.275	27/12	5/9	0.054	0.048	84/89
CPV, n(%)	37(39.8)	13(48.1)	0.508	4(10.3)	10(71.4)	< 0.001	0.062	64(37.0)

*Abbreviation BMI* Body Mass Index, *COPD* chronic obstructive pulmonary disease, *KL-6* Krebs von den Lungen-6, *P/F ratio* artery PO2 divided by the fraction of inspired oxygen, *NLR* neutrophil lymphocyte ratio, *CRP* C-reactive protein, *IL-6* serum interleukin 6 concentration, *ALT* Alanine transaminase, *LDH* lactate dehydrogenase, *SCr* serum creatinine, *BUN* Urea nitrogen, *RSG* Radiological Severity Grade, *GGO* grand glass opacity, *CPV* crazy paving pattern.*, cancers referred to any malignancy

We analysed the correlation between KL-6 level and RSG or CPV sign, and showed that KL-6 was positively correlated with RSG (*r* = 0.734, *P* < 0.001) and CPV (*r* = 0.387, *P* < 0.001) in the development group. The KL-6 level in patients with a high RSG (≥ 3) was higher than that in patients with a low RSG (< 3) (872(577–1039) vs. 318 (210–455), U/ml, *P* < 0.001) in development group. And the KL-6 level in patients with CPV sign was higher than that in patients without CPV signs (591(398–905) vs. 325(207–567), U/ml, *P* < 0.001) in the development group. Meanwhile, it showed that the level of IL-6 was higher in validation group when compared with development group (40.54(13.90-98.58) vs. 21.46 (7.35–56.62), pg/ml, *P* = 0.030); and the ratio of pure GGO in radiography in validation group was higher when compared with development group (32/53, 60.4% vs. 52/120, 43.3%, *P* = 0.048).

### Cox regression analysis

The 6 variables were significantly correlated with 28-day mortality including disease severity, serum KL-6, P/F ratio, NLR, IL-6 and RSG in both the development group and validation group (*P* < 0.05, Table 1). Univariate Cox regression analysis was performed, and showed that these variables significantly different (*P* < 0.05, Table 2). Then, we further performed a multivariable Cox regression analysis based on KL-6 and RSG. The hazard ratios of KL-6 and RSG were 1.001 (95% CI:1.000-1.002, *P* = 0.023) and 2.191 (95% CI:1.253–3.832, *P* = 0.006), respectively. It indicated that KL-6 and RSG were independent risk factors.

**Table 2:** Univariate cox regression for 28-day mortality in hospitalization in the development group

Variables	HR	95%CI	*p* value
Disease severity (severe/critical)	12.128	2.864–51.347	0.001
Serum KL-6 concentration, U/ml	1.002	1.001–1.003	< 0.001
P/F ratio	0.986	0.981–0.990	< 0.001
NLR	1.029	1.016–1.041	< 0.001
IL-6, pg/ml	1.001	1.001–1.002	< 0.001
RSG	3.200	2.066–4.955	< 0.001

*Abbreviation HR* hazard ratio, *CI* confidence index, *KL-6* Krebs von den Lungen-6, *P/F ratio* artery PO2 divided by the fraction of inspired oxygen, *NLR* neutrophil lymphocyte ratio, *IL-6* serum interleukin 6 concentration, *RSG* Radiological severity grade

### Nomogram construction and calibration

**Fig. 2:**
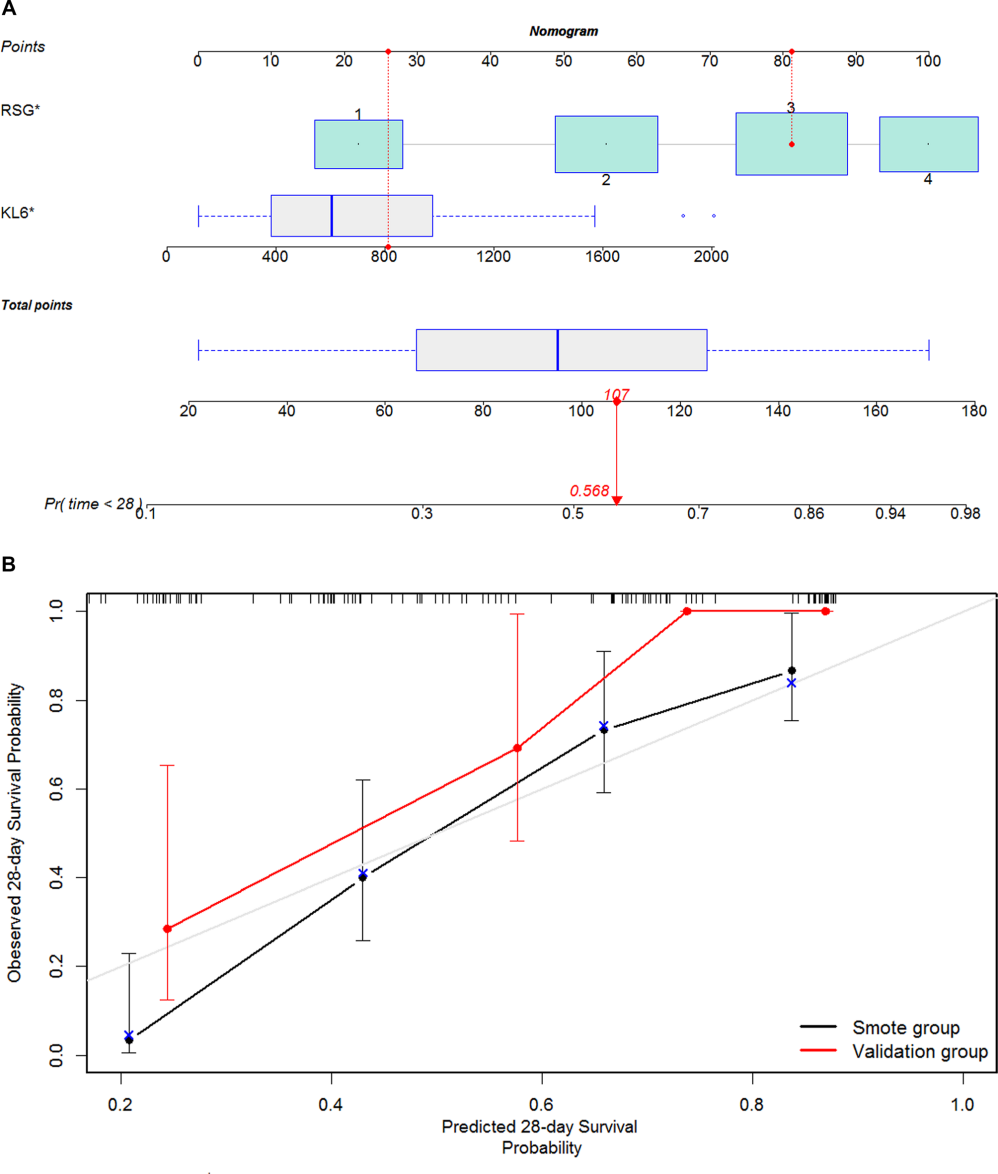
Nomogram model and calibration curve. (1) KL-6 and RSG were used to develop a nomogram model by SMOTE strategy to predict 28-day mortality in COVID-19 patients. For example (indicated by a solid red circle and arrow), the KL-6 and RSG results of an admitted patient were 810U/ml and grade 3, respectively. The points for his/her KL-6 and RSG were approximately 26 and 81 respectively. Hence, the total point for this patient was 107, which indicated a probability of 0.568 for the development of death events during 28 days after admission. (2) Calibration curve of the nomogram in the smote group and validation group, in terms of agreement between the predicted risk and actual observed outcomes. KL6, Krebs von den Lungen-6; RSG, radiological severity grade

SMOTE strategy was used to produce synthetic examples to overcome the problem of class imbalance for the development group. A nomogram predictive of 28-day mortallity was developed based on KL-6 and RSG with SMOTE strategy synthetic examples as smote group (Fig. 2). The C-index of the nomogram in the smote group and validation group was 0.687 (95% CI: 0.613–0.761) and 0.872 (95% CI: 0.798–0.946), respectively. The performance of the nomogram model was analysed by calibration curve (Fig. 2).

### Predictive performance of the KL-6, RSG and combined scores

**Fig. 3:**
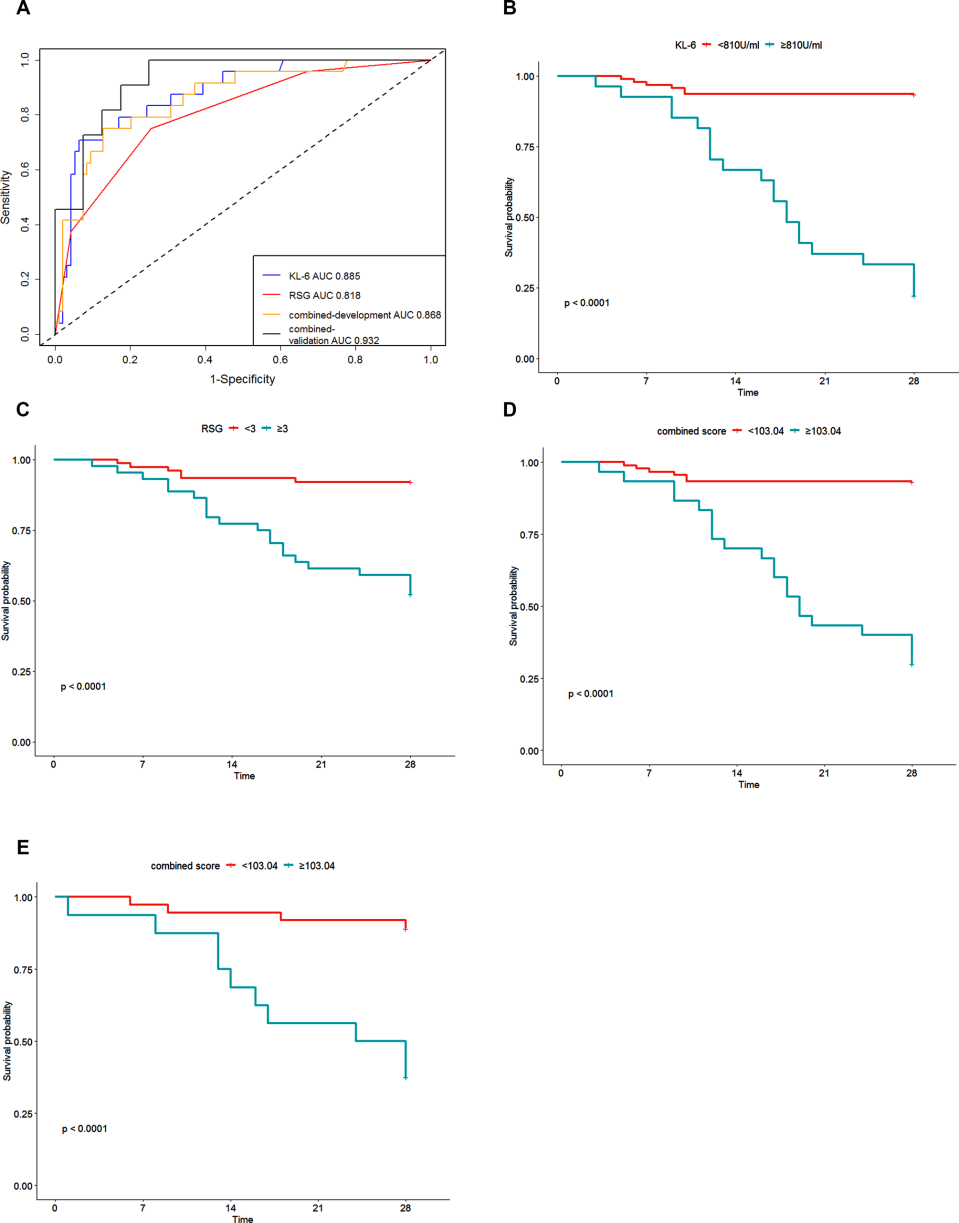
ROC curve analysis using KL-6, RSG and combined score for predicting the 28-day mortality, and Kaplan-Meier curves of 28-day survival in patients according to binary risk group stratification. (1) KL-6 yielded an AUC of 0.885 (95% CI: 0.804–0.952), while RSG yielded an AUC of 0.818 (95% CI: 0.711–0.899). The combined score in both development group and validation group yielded AUCs of 0.868 (95% CI: 0.776–0.942) and 0.932 (95% CI: 0.862–0.997), respectively. (2) K-P curve of KL-6 (low risk: <810 U/ml; high risk: ≥ 810 U/ml), (3) K-P curve of RSG (low risk: <3; high risk: ≥3), (4) K-P curve of combined score in the development group (low risk: < 103.04; high risk: ≥ 103.04), (5) K-P curve of combined score in the validation group (low risk: < 103.04; high risk: ≥103.04). *KL6* Krebs von den Lungen-6, *RSG* radiological severity grade, *K-P* Kaplan-Meier

Survival ROC curve analysis was applied to analyse the predictive performance of KL-6, RSG and the combined score (Fig. 3). The optimal cut-offs and relevant sensitivity and specificity are listed in Table 3. The combined score in the development group yielded an AUC of 0.868 (95% CI: 0.776–0.942, *P* < 0.001) with 46.2% sensitivity and 99.5% specificity. The combined score in the validation group yielded an AUC of 0.932 (95% CI: 0.862–0.997, *P* < 0.001) with 76.9% sensitivity and 90.0% specificity. In the development group, the AUC of KL-6 was higher when compared with the AUC of RSG (0.885 vs. 0.818, *P* = 0.024), the AUC of combined score was higher when compared with that of RSG as well (0.868 vs. 0.818, *P* = 0.004), while the AUC of KL-6 didn’t show difference when compared with the AUC of combined score (0.885 vs. 0.868, *P* = 0.234).

**Table 3:** Predictive performance of KL-6, RSG and combined score for 28-day mortality in COVID-19 patients

Variables	Cut-off		AUC(95%CI)		Sensitivity		Specificity		*P* value			
KL-6, U/ml			810		0.885(0.804–0.952)		73.1%		93.6%		< 0.001	
RSG			3		0.818(0.711–0.899)		76.9%		74.5%		< 0.001	
Combined scores (development group)			103.04		0.868(0.776–0.942)		46.2%		95.0%		< 0.001	
Combined scores (validation group)			103.04		0.932(0.862–0.997)		76.9%		90.0%		< 0.001	

*Abbreviation KL-6* Krebs von den Lungen-6, *RSG* Radiological Severity Grade, *AUC* the area under the curve, *CI* confidence interval

On the basis of the optimal cut-off based on the ROC curve analysis, all indicators were transformed into binary variables, and risk ratios were calculated. The risk ratios of KL-6, RSG, and combined scores in the development group and validation group were associated with a higher incidence of 28-day mortality, with risk ratios of 17.858 (95% CI: 7.130-44.729, *P* < 0.001), 7.279 (95% CI: 2.932–18.069, *P* < 0.001), 14.499 (95% CI: 5.810-36.184, *P* < 0.001), 7.873 (95% CI: 2.454–25.261, *P* = 0.001), respectively.

### Kaplan - Meier curve of subgroups stratified by KL-6, RSG and combined scores

All 173 inpatients were followed in this retrospective study. The primary endpoint occurred in 41 inpatients (23.7%). Patients were categorized into two subsets according to the optimal cut-off value of KL-6, RSG and combined score in the development group (Table 3). Kaplan-Meier curves showed that differences between the two subsets were statistically significant (Fig. 3) in the development group. In the validation group, the result showed that patients with a high combined score had poor outcomes compared to those with a low combined score (Fig. 3).

## Discussion

COVID-19 is still a global health threat that can develop into severe/critical illness resulting in high morbidity and mortality worldwide. Identifying the predictors for severe illness or mortality has been recognized as one of the most important issues for controlling COVID-19 [13]. Older age has been recognized as a risk factor for mortality in COVID-19 patients [6, 14]. Older patients are usually associated with a high prevalence of comorbidities, such as hypertension, diabetes; and decreased reserve capacity of vital organs, which may lead to increased frailty and a higher risk of a poor outcome. In our study, the majority of patients (87.9%) were above the age of 60 years, and their median age was 74 (range, 23–95 years). Therefore, the age of non-survivals was somehow higher than survivors although without statistical significance.

KL-6 is a mucin like glycoprotein distributed mainly on the surface of type II alveolar epithelial cells (AECs) and respiratory bronchiolar epithelia cells [15], and it has been well-explored in idiopathic pulmonary fibrosis (IPF) and ARDS patients, and is recognized as one of the specific biomarkers of AECs damage [16]. Recently, Deng et al., declared that serum KL-6 levels in COVID-19 patients were higher than those in healthy patients; serum KL-6 levels in severe/critical COVID-19 patients were especially higher than those in mild patients (median 898.0 vs. 452.1 U/ml, *P* < 0.001) [17]. They also found that KL-6 increased from symptom onset, peaked within approximately a month, and then gradually decreased. It took less time to reach a higher level in severe/critical patients than in mild patients. Meanwhile, serum KL-6 was linearly correlated with computed tomography lung lesion areas. Arnold et al. [18] compared baseline KL-6 levels in patients with COVID-19 and a cohort of IPF, and found that the KL-6 level showed no significant difference between severe COVID-19 and IPF, while KL-6 levels were significantly lower in mild or moderate COVID-19 when compared with IPF. This result indicated that patients with severe COVID-19 presented with a higher baseline KL-6 level than mild or moderate COVID-19. Frix et al. [19] investigated a cohort of 83 COVID-19 patients and found that serum KL-6 was a prognostic biomarker of disease activity and fibrosis in COVID-19; however they did not find any correlation between KL-6 levels and admission to intensive care or mortality. Our results showed that KL-6 levels were much higher in non-survivor than in survivors (Table 1, *P* < 0.001). For those with high KL-6 (≥ 810U/ml), the 28-day survival rate was lower, than that for those with lower KL-6 (< 810U/ml) (Fig. 3).

Chest imaging, especially HRCT is used for the early diagnosis of COVID-19 to evaluate lung involvement [20–23]. The CT lung lesion area was found to be linked to COVID-19 progression [24], severe lung involvement was more common in patients presenting with CPV, and patients with GGOs in moderate lung involvement were more likely to recover faster, than those with CPV [25]. In our study, patients with severe lung involvement were relevant with 28-day mortality event, which is in agreement with a previous study [20]. The RSG yielded an AUC of 0.818 (95% CI: 0.711–0.899, *P* < 0.001), which indicated that patients with a higher RSG (grade ≥ 3) would have a poor outcome (Fig. 3). A total of 56.9% (37/65) patients had a higher RSG (grade 3 and 4), accompanied by the CPV sign. In contrast, 25.0% (27/108) of patients had a lower RSG (grade 1 and 2), accompanied by the CPV sign. This result indicated that severe lung involvement was more likely to coexist with the CPV sign.

We further analysed the correlation between KL-6 level and RSG or CPV sign, and showed that high KL-6 level was linked to RSG and to CPV (*P* < 0.001) in the development group. Patients with high KL-6 levels were more likely to present with an RSG greater than grade 3 or the CPV sign. These results indicated that KL-6 correlated with lung involvement.

This study has limitations mainly due to its retrospective nature, single-center design and selection bias. So, the model was developed in this study should be interpreted with caution. In addition, the primary endpoint was 28-day mortality after admission, the effect of applied interventional extracorporeal therapies such as extracorporeal membrance oxygenation (ECMO） on survival was not studied as this was not an endpoint of this study. Despite these limitations, we found that KL-6 and RSG were potential risk factors of 28-day mortality in COVID-19 patients with pneumonia. Certainly, different factors affecting mortality were identified based on the various studies’ designs thus far. From that point of view, more prospective and multi-center studies are needed to assess the possible factors affecting mortality in patients with COVID-19 and clarify the understanding mechanisms to improve the management of patients and to help in the development of new forms of treatment.

## Conclusions

In summary, our results suggested that the nomogram and combined score derived from KL-6 and RSG, might be a potential method for evaluating 28-day mortality in COVID-19 patients with pneumonia. Patients with higher KL-6 levels and higher RSG should be carefully considered. However, our results should be interpreted with caution and validated in longitudinal studies in a larger and multi-center cohort.

## Data Availability

The datasets used and/or analysed during the current study available from the corresponding author on reasonable request.

## Abbreviations

COVID-19: Coronavirus disease 2019
HRCT: high resolution computed tomography
BMI: Body Mass Index
COPD: chronic obstructive pulmonary disease
KL-6: Krebs von den Lungen-6
AECs: type II alveolar epithelial cells
P/F ratio: artery PO2 divided by the fraction of inspired oxygen
NLR: neutrophil lymphocyte ratio
CRP: C-reactive protein
IL-6: serum interleukin 6 concentration
GPT: glutamate pyruvate transaminase
LDH: lactate dehydrogenase
SCr: serum creatinine
BUN: Urea nitrogen
GGO: ground glass opacity
CPV: crazy paving pattern
AUC: area under the curve
CI: confidence interval
ECMO: extracorporeal membrance oxygenation
RSG: Radiological severity grade
IPF: idiopathic pulmonary fibrosis
ARDS: acute respiratory distress syndrome
ILDs: interstitial lung diseases
IQR: interquartile range
ROC: receiver operating characteristic
HR: hazard ratio
OR: odds ratio
SMOTE: synthetic minority oversampling technique
